# Evaluation of a Collaborative Practice Agreement Intervention to Improve Access to HIV Prevention Services in Southeastern US Pharmacies: Protocol for a Hybrid Type 1 Effectiveness-Implementation Study

**DOI:** 10.2196/76058

**Published:** 2026-05-11

**Authors:** Chante Hamilton, Alexis Hudson, Christina Chandra, Seth Zissette, Rachel Rothman, Daniel I Alohan, Donald G Klepser, Alvan Quamina, R Wayne Woodson, David Holland, Natalie D Crawford

**Affiliations:** 1 Department of Behavioral, Social, and Health Education Sciences, Rollins School of Public Health, Emory University, 1518 Clifton Road, NE, Atlanta, GA, 30322, United States, 1 4047129445; 2 Department of Epidemiology, Rollins School of Public Health, Emory University, Atlanta, GA, United States; 3 Georgia Commission on Family Violence, Atlanta, GA, United States; 4 College of Pharmacy, Department of Pharmacy Practice and Scien, University of Nebraska Medical Center, Omaha, NE, United States; 5 National Aids Education and Services for Minorities, Atlanta, GA, United States; 6 Mercy Care, Atlanta, GA, United States

**Keywords:** HIV prevention, PrEP, pre-exposure prophylaxis, collaborative practice agreements, pharmacy, implementation science

## Abstract

**Background:**

The United States (US) Southeast experiences a disproportionate burden of HIV, compounded by limited access to pre-exposure prophylaxis (PrEP) services due to systemic barriers, such as stigma, medical mistrust, and restrictive pharmacy policies. Collaborative practice agreements (CPAs) between pharmacists and clinicians represent a promising strategy to improve PrEP accessibility through pharmacy-based services.

**Objective:**

The Collaborative Agreement–based PrEP Using Pharmacies (CAP-UP) study aims to evaluate the development, implementation, and effectiveness of a CPA template and training resources to enhance pharmacy-based PrEP initiation and adherence in the Southeastern US.

**Methods:**

The CAP-UP study uses a hybrid type 1 effectiveness-implementation design guided by integrated implementation science frameworks. It includes three phases: (1) exploration, using online surveys and in-depth interviews to identify barriers and facilitators to CPA implementation; (2) preparation, developing and refining a CPA template and training resources; and (3) implementation, piloting the CPA template in 10 community pharmacies within high-HIV-prevalence neighborhoods.

**Results:**

The CAP-UP study was funded in October 2023 by the National Institutes of Health through the Center for AIDS Research (grant P30-AI050409-25S1) as an Ending the HIV Epidemic supplement. Recruitment launched in November 2023 and is actively ongoing, with 264 pharmacy staff having completed surveys as of January 2026. The study aims to recruit a total of 300 participants for surveys and conduct 68 in-depth interviews by February 2026.

**Conclusions:**

Expanding HIV prevention services through community pharmacies represents a practical and impactful strategy for addressing inequities in HIV prevention that advances health equity. By leveraging implementation science frameworks, our study explores a novel approach to integrating HIV prevention services in pharmacies located in areas with high HIV prevalence. Findings from this work will contribute to the evidence base needed to optimize pharmacy-based PrEP delivery and support the national goal of Ending the HIV Epidemic in the US.

## Introduction

### Background

The HIV epidemic continues to affect marginalized groups disproportionately, particularly in the United States (US) Southeast, which accounted for 49% of HIV infections in 2022 [1]. Pre-exposure prophylaxis (PrEP) is an essential intervention in reducing the risk of HIV transmission. When taken as a daily pill as prescribed, PrEP decreases the likelihood of infection from sexual contact by approximately 99% and from injection drug use by at least 74%. This study focuses on the implementation of daily oral PrEP through collaborative practice agreements (CPAs), though injectable PrEP may be considered in future efforts [2]. The expansion of PrEP usage is globally recognized as a critical component of a comprehensive approach to reducing new HIV transmissions [3-8]. Despite the proven benefits, the US Southeast faces significant challenges in PrEP accessibility, as highlighted by its 2022 PrEP-to-need ratio (PnR) of 10.0, meaning there are 10 PrEP users for every person diagnosed with HIV. In comparison, the West, Northeast, and Midwest have higher PnRs of 16.2, 21.5, and 15.9, respectively [9]. This low PnR signals an unmet need for PrEP fueled by a backdrop of systemic barriers, including lack of proximity to PrEP providers [10-14], low health insurance coverage, and high cost of medical care [12,14-19], medical mistrust [14,16,20-24], stigma [14,16,21-26], and limited PrEP knowledge among providers and populations at risk of contracting and transmitting HIV [15,16,27]. These barriers continue to hinder PrEP access, uptake, and adherence among individuals at risk from sexual exposure and injection drug use [28-31].

### Pharmacies Are Key Access Points for HIV Prevention Services

Research has demonstrated that enhancing support for state and local initiatives to increase pharmacy-based access to PrEP can significantly improve the HIV care continuum [32,33]. Pharmacies, as primary access points for prescriptions, can play a crucial role in PrEP acquisition and adherence [33]. Over 89% of Americans reside within 5 miles of a pharmacy, and many consumers frequently visit pharmacies during off-clinic hours, such as weekends, evenings, and holidays [34]. Pharmacies are ideally situated within neighborhoods experiencing high rates of HIV, which are often communities with higher proportions of Black residents and lower income levels [35].

Pharmacists are viewed as trusted sources of health care by community members [33-36]. There is a growing body of evidence showing that community pharmacies can engage with high-risk populations to provide HIV testing [37-40] and counseling to reduce HIV risk behaviors [41-43]. As members of the community that they provide services in, pharmacists have a strong rapport with community members and the potential to reduce stigma when offering HIV services [44-46] in a nonstigmatized setting and concurrently with other nonstigmatized disease screenings [37,38,47]. They also report high willingness and ability to be trained to screen for and dispense PrEP [47-51]. Unfortunately, pharmacy-based PrEP uptake in the Southeastern US faces significant policy-driven barriers that hinder pharmacists’ ability to initiate and manage PrEP care effectively.

### CPAs: A Viable Strategy for Integration

Currently, pharmacists’ scopes of services are primarily defined by national- and state-level policies, with many already delivering essential services, such as medication, immunizations, and primary prevention screenings [52,53]. In the US Southeast, local policies often restrict pharmacists from prescribing PrEP, requiring formal linkages with clinicians who can provide telehealth prescriptions [54,55]. Pharmacists and PrEP-prescribing clinicians in every state in the US Southeast can establish linkages called CPAs, which represent a viable pathway to further integrating pharmacists into HIV care [56,57]. CPAs allow pharmacists to work directly with clinicians to provide comprehensive health services, including HIV prevention services, such as PrEP prescribing, HIV testing, and follow-up care [58,59]. This model has the potential to increase the availability of PrEP services and formalize the partnership between pharmacists and clinicians, ensuring that high-risk populations can be effectively and consistently linked to HIV care [60-62]. Furthermore, little is known about provider sentiments toward addressing this barrier. Thus, examining provider willingness to establish CPAs and the various implementation needs for PrEP care linkage in the US Southeast is imperative to maximize this pharmacy-based strategy to Ending the HIV Epidemic (EHE) [7].

### Conceptual Frameworks Guiding CPA Development and Implementation

**Figure 1.**
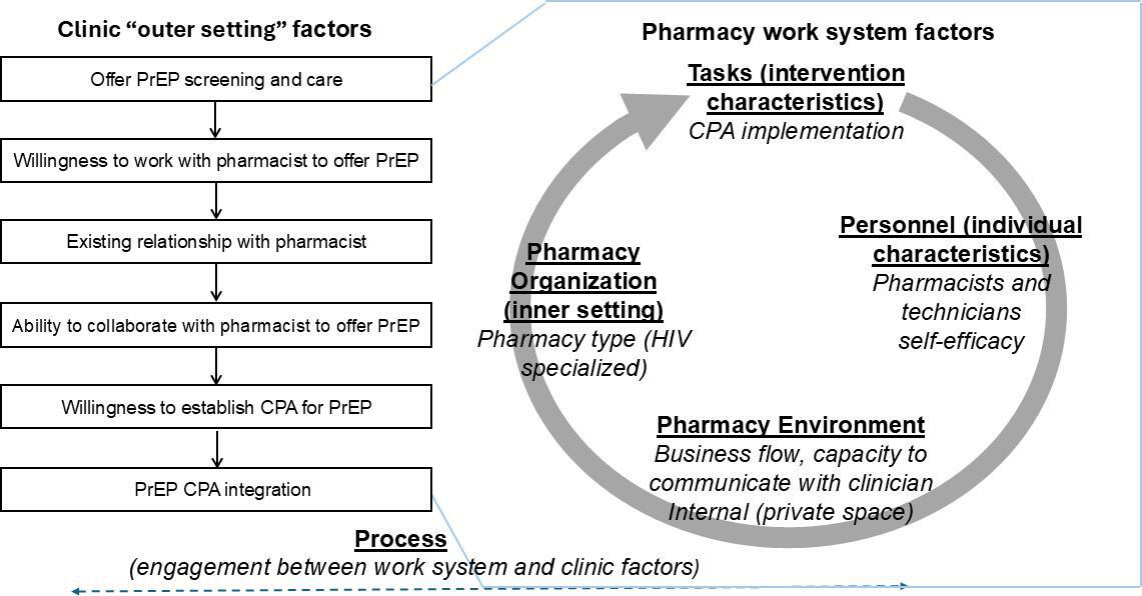
Conceptual framework for establishing a pharmacist-clinician CPA guided by the Consolidated Framework for Implementation Research and Systems Engineering Initiative for Patient Safety. CPA: collaborative practice agreement; PrEP: pre-exposure prophylaxis.

Improving collaboration between pharmacists and PrEP-prescribing clinicians requires a thorough understanding of the factors influencing the establishment of CPAs. This study examines policy-level constraints, pharmacy workflows, and clinician engagement to identify barriers and facilitators shaping the integration of PrEP into community pharmacies. To guide this process, we will apply the Exploration, Preparation, Implementation, Sustainment (EPIS) framework, which offers a structured approach to intervention development [63]. The EPIS framework includes 4 stages: Exploration, to identify barriers and facilitators of CPA adoption through stakeholder input; Preparation, to create and adapt a CPA template that supports pharmacy-based PrEP services; Implementation, to pilot the use of CPAs in community pharmacy settings; and Sustainment, to evaluate the long-term factors that influence CPA scalability and effectiveness. This study also integrates the Consolidated Framework for Implementation Research (CFIR), which systematically evaluates 5 domains critical to implementation: intervention characteristics, external influences, internal pharmacy operations, individual roles, and implementation processes [64]. Additionally, the Systems Engineering Initiative for Patient Safety (SEIPS) model will be used to assess how pharmacy work systems, processes, and outcomes interact throughout the implementation of CPAs. SEIPS, often used in pharmacy interventions, such as medication therapy management, emphasizes designing work systems to optimize care delivery [65]. Figure 1 illustrates the conceptual framework for establishing a pharmacist-clinician CPA, integrating CFIR and SEIPS to highlight key factors influencing implementation. By integrating these frameworks, this study aims to provide a detailed understanding of the barriers and opportunities for CPA implementation to improve access to HIV prevention services through pharmacies.

### Objectives

The primary goal of the Collaborative Agreement–based PrEP Using Pharmacists (CAP-UP) study is to develop and evaluate a pharmacist-clinician CPA template and training program to improve access to pharmacy-based PrEP initiation and adherence services in the US Southeast. This study aims to (1) identify and evaluate policy-, pharmacy-, and clinician-level barriers and facilitators to implementing CPAs for PrEP services in community pharmacies, providing actionable insights to enhance PrEP delivery systems; (2) design and test a flexible CPA template that can be adapted across diverse pharmacy settings to facilitate the integration of HIV prevention services and equip pharmacists with the tools needed to deliver comprehensive care; and (3) pilot and assess the implementation of pharmacist-clinician CPAs in community pharmacies, focusing on effectiveness and implementation outcomes, including acceptability, feasibility, and care linkage [64,65]. We will use a hybrid type 1 design that allows for the simultaneous evaluation of implementation and effectiveness outcomes, offering valuable evidence for real-world CPA adoption [66]. In the following sections, we outline the protocol for each aim of CAP-UP.

## Methods

### Study Design

**Figure 2.**
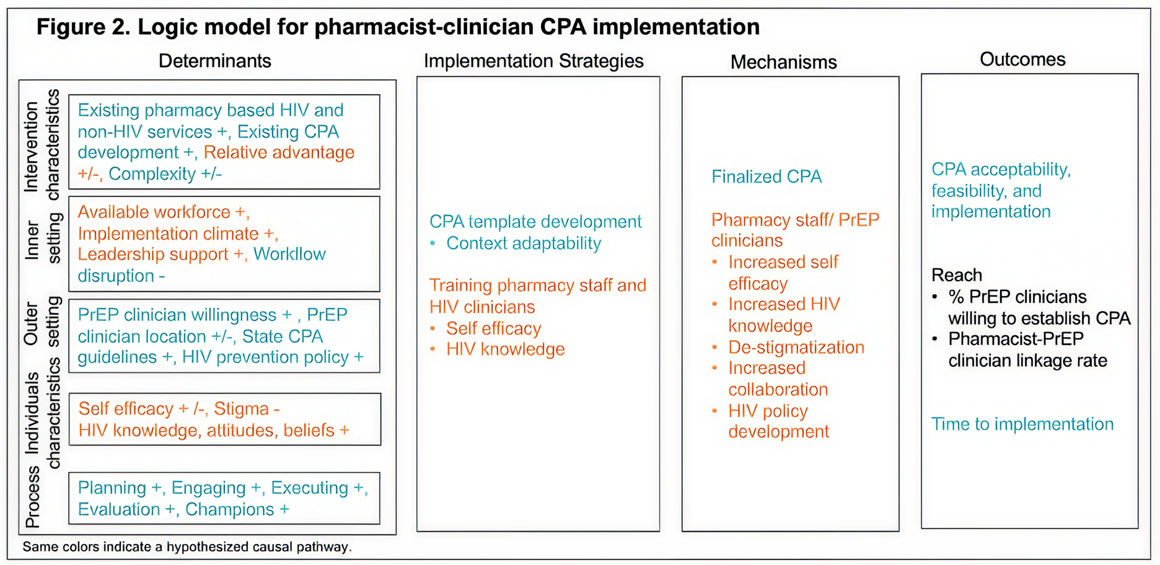
Logic model for pharmacist-clinician CPA implementation. Same colors indicate a hypothesized causal pathway. CPA: collaborative practice agreement; PrEP: pre-exposure prophylaxis.

The CAP-UP study uses a mixed methods, hybrid type 1 design to test the effectiveness and evaluation of establishing CPAs between pharmacists and clinicians for delivering PrEP services in pharmacies. Drawing on the EPIS framework, the study is conducted in three phases: (1) an exploration phase with a mixed methods assessment of barriers and facilitators to CPA establishment, including online surveys with pharmacy staff from across the US Southeast and in-depth interviews (IDIs) with pharmacy policy decision-makers, pharmacy staff, and PrEP-prescribing clinicians in Georgia; (2) a preparation phase to develop a CPA template and resources for pharmacists and clinicians to formalize partnerships for PrEP care provision; and (3) an implementation and sustainment phase to test the CPA template at 10 community pharmacies in neighborhoods with high baseline HIV prevalence, to evaluate its integration in pharmacy and clinic settings. We propose testing a logic model for scaling pharmacy-based PrEP services using pharmacist-clinician CPAs, as outlined in Figure 2.

### Ethical Considerations

Ethics approval was obtained from the Emory University Institutional Review Board (STUDY00006422). All procedures involving human participants will adhere to ethical principles and federal regulations for the protection of human participants. Informed consent will be obtained from all participants prior to participation. For the web-based survey, participants will review and electronically agree to a consent form before beginning the survey. For the qualitative interviews, verbal consent will be obtained and documented at the start of each interview. All data will be deidentified prior to analysis. Survey responses will be collected through a secure, password-protected platform, and interview transcripts will have personal identifiers removed during transcription. Study data will be stored on encrypted servers accessible only to authorized research staff. Participants completing the survey will receive a US $20 electronic gift card, and stakeholders participating in interviews will receive a US $50 electronic gift card as compensation. No identifiable images of participants will be included in this publication or any future study materials.

### Phase 1: Exploration Phase

The primary purpose of the exploration phase is to establish an understanding of the policy-, pharmacy-, and clinician-level barriers and facilitators of integrating CPAs into community pharmacies to expand PrEP access.

### Recruitment

#### Pharmacy staff

To recruit pharmacy staff, including pharmacists and technicians, for online surveys (n=300), we obtained an exhaustive list of registered pharmacies in the US from the National Council for Prescription Drug Programs database. This list provides information about pharmacies, including names, addresses, phone numbers, National Provider Identifier numbers, and pharmacy-type classifications (eg, independent, chain, and compounding). We mapped the geographic locations of pharmacies to identify their presence within National Institutes of Health priority jurisdictions in the Southeast to inform recruitment [67]. Pharmacies included in this list will be called randomly to determine their willingness to participate in an online survey. From the online survey respondents, we will purposively select 50 participants for IDIs to gain a deeper understanding of their experiences and perspectives. Pharmacy staff will be recontacted based on their location and whether their pharmacy provides non-HIV-related screening services to confirm their willingness to participate in the interviews.

#### Board of Pharmacy Members

To recruit pharmacy policy decision-makers, we will compile a list of individuals based on the guidance from expert pharmacy collaborators who will reach out to each individual to introduce the study and its objectives. Following this, we will request an interview with them and ask them to participate in our study. Our goal is to recruit 2 decision-makers from each state to participate in the study.

#### PrEP-Prescribing Clinicians

PrEP-prescribing clinicians will also be recruited in phase 1 to participate in IDIs. Clinicians within the study region will be identified using PrEP Locator [68], a national directory of providers of HIV pre-exposure prophylaxis (PrEP) in the US, along with existing clinical partner networks and referrals from collaborating stakeholders; clinicians will be contacted via email to introduce the study and invite participation. Clinicians who express interest will be scheduled for an interview to provide perspectives on CPA development, PrEP service delivery, and pharmacist-clinician collaboration within the Southeastern US context.

### Data Collection

#### Pharmacy Staff Survey

The 15-minute online survey for pharmacy staff will assess the readiness of pharmacies to integrate CPAs for HIV prevention services. Guided by the CFIR and SEIPS implementation science frameworks, the survey will evaluate several dimensions of readiness, including self-efficacy in providing CPAs and PrEP services, level of CPA and HIV-related training, prior experience with pharmacist-physician collaboration, availability of HIV-related information within the pharmacy, time availability, knowledge of CPA and HIV services, attitudes and beliefs about these services, and levels of HIV-related stigma [69].

#### Key Stakeholder IDIs

The interview guide measures will address a range of topics across 3 key domains: general public health and HIV/AIDS policy, pharmacy-clinician CPA implementation, and pharmacy-based HIV prevention services. Informed by CFIR and SEIPS, specific questions will explore support for HIV prevention services and CPAs, policies shaping HIV service delivery, referral and monitoring processes within pharmacies, potential barriers to implementing CPAs, knowledge of HIV epidemiology and related risk behaviors, and factors affecting the sustainability of pharmacy-based PrEP services. Interview guides will be tailored for each stakeholder group to reflect their distinct roles in CPA development and HIV prevention service delivery. Pharmacy staff interviews will explore workflow processes, operational capacity, referral and monitoring procedures, and perceived barriers and facilitators to CPA implementation within the pharmacy setting. PrEP-prescribing clinician interviews will focus on pharmacist-clinician collaboration, prescribing practices, coordination of PrEP care, and perspectives on integrating pharmacy-based PrEP services. Board of pharmacy interviews will address regulatory frameworks, state-level policies influencing CPA use, and broader public health and HIV/AIDS policy considerations. Across all groups, interviews will examine factors influencing the sustainability of pharmacy-based PrEP services and support for HIV prevention within community settings.

#### Key Stakeholder Compensation

Pharmacy staff who complete the online survey will be sent a US $20 electronic gift card via email. Key stakeholders will be compensated with a US $50 electronic gift card for IDIs.

### Planned Analyses

#### Online Survey and Data Cleaning and Analysis

Quantitative analyses will be conducted on the online survey data completed by pharmacy staff in phase 1. PrEP-prescribing clinicians do not complete a quantitative survey; therefore, their contributions to phase 1 are limited to qualitative interview data, which will be analyzed separately. Survey data will be cleaned by creating new variables and consolidating response options where needed. Exploratory data analysis will be conducted using SAS (SAS Institute Inc) or R (R Foundation for Statistical Computing) programming to calculate descriptive statistics, including means, medians, percentages, proportions, SDs, and measures of skewness and kurtosis. Group differences for continuous variables will be analyzed using *t* tests, with normality transformations applied if necessary, or rank-based tests for nonnormally distributed data. For categorical variables, comparisons between groups will be made using *χ*^2^ tests, exact tests, and 95% CIs to support interpretation.

To examine factors associated with CPA implementation readiness, regression modeling will account for potential mediators and confounders, such as years of pharmacy experience. Outliers will be evaluated using sensitivity analyses, comparing results with and without outliers. Nonparametric tests will be used when appropriate. Relationships between confounders and outcomes will be explored through *t* tests or rank-based tests for continuous variables and exact tests for categorical variables. Significant bivariate relationships will be incorporated into multivariate models, using linear regression for continuous outcomes and logistic regression for binary outcomes.

#### Key Stakeholder Data Cleaning and Analysis

IDIs will be digitally recorded, transcribed verbatim by a professional service, and analyzed using reflexive thematic analysis. A combination of deductive coding, guided by CFIR and SEIPS implementation science frameworks, and inductive coding for emergent themes will be applied. Two analysts will independently code transcripts to identify patterns and develop a preliminary codebook. This process will generate detailed narratives on stakeholders’ perspectives regarding CPA implementation for PrEP services. Analysts will maintain reflexivity by documenting potential biases and assumptions through memos throughout the analysis to enhance validity and transparency. The research team will conduct data analysis using MAXQDA Analytics Pro (VERBI Software).

### Phase 2: Preparation Phase

The primary goal of the preparation phase is to develop a CPA template that is adaptable to various states in the US Southeast.

#### CPA Template Development

The data collected will be thoroughly analyzed to guide the creation of a CPA template. An initial CPA will be designed to include essential elements, such as the scope of the agreement and its legal and administrative requirements (Table 1). Variations of the CPA template will then be tailored to address different pharmacy, clinician, and policy contexts, such as existing HIV prevention services and regional HIV-related policies. A Georgia-specific CPA template will be finalized for use in the next study phase, with iterative revisions informed by data from the facilitated implementation in phase 3 to ensure its practicality and acceptance. This adaptive development approach, commonly used in early-phase clinical trials, supports continuous improvement and efficient integration of findings [70]. Detailed records of all CPA template development and revisions will be maintained, and the final versions of the CPA template will be made publicly accessible through the Emory Center for AIDS Research website.

**Table 1.** Components of CPAs^a^ for pharmacy-based PrEP^b^ services.

Domain/component	Description	Application to CPA implementation
Scope of agreement		
Patient inclusion criteria	Defines the specific patient population eligible for services under the CPA	Identify patients at high risk of HIV or those seeking PrEP initiation and adherence support
Patient care functions authorized	Details the specific tasks the pharmacist is allowed to perform	Authorize activities such as initiating or modifying PrEP therapy, ordering/interpreting lab tests, performing physical assessments, and providing adherence counseling
Modify existing therapy	Permits adjustments to existing treatments within the scope of the CPA	Allow pharmacists to modify PrEP regimens when appropriate based on clinical guidelines
Initiate new therapy	Allows pharmacists to start new PrEP therapy for eligible patients	Enable pharmacists to prescribe and initiate PrEP treatment directly under the CPA
Laboratory testing	Authorizes ordering, performing, or interpreting lab tests as necessary for PrEP care	Specify laboratories for HIV, renal function, or STI^c^ testing critical to PrEP initiation and monitoring
Legal components		
Authority and purpose	Establishes the legal framework and purpose of the CPA	Define the CPA’s focus on improving PrEP access and adherence while ensuring compliance with state regulations
Liability insurance	Requires all parties to maintain appropriate insurance coverage	Ensure pharmacists and clinicians have professional liability insurance specific to PrEP services
Informed consent	Mandates obtaining patient consent for services provided under the CPA	Develop standardized procedures for documenting patient consent for PrEP initiation and care
Review and validity	Specifies the duration, review process, and terms for rescinding or altering the CPA	Establish timelines for CPA review and processes for amendments or terminations as needed
Signatures	Requires signatures from all parties involved in the CPA	Formalize agreements with signatures from participating pharmacists and clinicians
Administrative components		
Training and education	Ensures all parties maintain up-to-date knowledge and skills	Provide ongoing training on PrEP guidelines, patient care, and CPA procedures
Documentation	Outlines requirements for recording CPA-related activities and patient interactions	Implement systems to document PrEP services, including initiation, counseling, and follow-ups
Communication	Establishes clear communication protocols between pharmacists, clinicians, and patients	Develop guidelines for regular updates, collaboration, and referrals between parties
Quality assurance	Implements measures to monitor and improve CPA-related services	Define metrics for evaluating CPA effectiveness and ensuring adherence to the protocol
Retention of records	Mandates secure and accessible record-keeping practices	Specify requirements for storing CPA-related data and patient records for compliance and accountability

a CPA: collaborative practice agreement.

b PrEP: pre-exposure prophylaxis.

c STI: sexually transmitted infection.

### Phase 3: Implementation and Sustainment Phase

The primary purpose of the implementation and sustainment phase is to test the implementation of the CPA template at 10 community pharmacies located in neighborhoods with high baseline HIV prevalence and evaluate the integration of this resource in the pharmacy and clinic settings.

### Recruitment

#### Pharmacy Sampling, Eligibility, and Enrollment

Ten independent community pharmacies in Georgia will be selected to pilot and evaluate the implementation of CPAs. Independent pharmacies, rather than chain or hospital pharmacies, will be targeted to streamline the approval process and manage any necessary adjustments internally during the study period. Pharmacies will be chosen based on their location, beginning with those identified in phase 1 within Georgia’s EHE priority jurisdictions. To focus on neighborhoods with the greatest need, we will prioritize census tracts where the population is over 70% Black. We will approach the lead or owning pharmacist at each pharmacy to gauge their interest in establishing a CPA for PrEP screening and care. Informed consent will be obtained from at least 1 pharmacist at each site, but participation will also be open to any additional pharmacists and technicians who express interest in contributing to the study.

#### Clinician Sampling, Eligibility and Enrollment

We will reach out to PrEP-prescribing clinicians in the Georgia EHE jurisdictions listed on PrEP locator [68] to determine their interest in establishing CPAs with pharmacies to support PrEP initiation and care. Initial contact will be made via email, introducing the study, explaining its objectives, and inviting their participation in the CPA development. If no response is received, we will follow up using a combination of phone and email, making up to 4 attempts on different days and times during working hours. Voicemails will be left during phone calls, and clinicians who remain unreachable after four attempts will be removed from the contact list.

Clinicians who express interest will be added to a publicly accessible list on the Emory CFAR website, serving as a resource for identifying those open to CPA partnerships. This finalized list will be used to match clinicians with pharmacies enrolled in the study, prioritizing those within a 10-mile radius. Pharmacists will review potential clinician partners based on publicly available information, such as patient population, years of practice, and educational background. While we aim to recruit as many clinicians as possible, matches will be determined by geographic proximity (including driving and public transportation) and pharmacist preferences. Some clinicians may be paired with multiple pharmacies based on these criteria.

#### Integration of the Pharmacist-Clinician CPA

The CPA created during the preparation phase will be shared with all participating pharmacists and clinicians. To ensure that the CPA is customized to each setting, a pharmacy community implementation advisor will be assigned to the pharmacy. The pharmacy community implementation advisor is an external expert trained in practice improvement and the implementation of clinical activities in a pharmacy setting. Previous research emphasizes the importance of adapting organizational strategies to fit existing workflows, making this tailored approach critical [71,72]. The implementation advisor will conduct 3 structured meetings per pharmacist–clinician partnership during CPA development and early implementation. This will include 1 individual meeting with the pharmacist, 1 individual meeting with the clinician, and 1 joint meeting to review, tailor, and finalize the standardized CPA template developed in phase 2. Additional follow-up meetings may be scheduled on an as-needed basis to support CPA refinement and implementation, informed by the ongoing assessment of feasibility and acceptability. To help integrate the CPA effectively into pharmacy workflows and clinician collaborations, the advisor will use 6 targeted strategies: developing quality improvement tools, organizing implementation team meetings, identifying and preparing champions, assessing readiness and addressing barriers, conducting local consensus discussions, and implementing cyclical small tests of change.

#### Pharmacist and Clinician Data Collection

Baseline interviews will be conducted during the initial 1-hour in-person CPA development and integration session facilitated by the pharmacy community implementation advisor. Follow-up interviews will be conducted 7 days after the CPA application is submitted. These interviews will collect data on key implementation outcomes, aligned with the SEIPS framework and detailed in Table 2, while also incorporating CFIR constructs to identify barriers and facilitators to CPA implementation.

**Table 2.** Variables to be collected among pharmacy staff and pre-exposure prophylaxis clinicians by the Systems Engineering Initiative for Patient Safety model.

Work system component	Description of variables
Personnel	Age, race/ethnicity, sex/gender, attitudes, willingness and perceptions of HIV prevention and CPA^a^ implementation, and HIV stigma
Organization	Years in practice, organizational work flow, average number of HIV and non-HIV service patients, and PPCI^b^
Pharmacy environment	Business flow, neighborhood characteristics (accessibility via public transportation, walkability, safety, physician and pharmacy access, and demographic characteristics)
Tasks	Ability to and perceptions of engaging clinicians about implementing a CPA, CPA self-efficacy, ability to communicate with clinician/pharmacist, and time spent communicating with pharmacist/HIV clinician

a CPA: collaborative practice agreement.

b PPCI: Physician-Pharmacist Collaborative Index.

#### Compensation for Pharmacy staff and PrEP Clinician Retention

Pharmacy staff will receive their average hourly rate (US $61 for pharmacists and US $19 for technicians) based on an estimate of 4 hours of their time implementing the CPA. Pharmacy staff as well as PrEP clinicians will also be compensated US $25 for each baseline and follow-up interview.

#### Effectiveness Outcomes

To assess effectiveness within this hybrid type 1 design, we will include client-level outcomes that reflect whether implementing pharmacist-clinician CPAs increases access to PrEP services in participating pharmacies. Pharmacies will report aggregate counts of clients who move through the key steps of the CPA-enabled PrEP pathway, including the number of individuals screened for PrEP eligibility, the number determined to be eligible, the number referred to or completing a consultation with the collaborating clinician, and the number who receive a PrEP prescription. The primary effectiveness outcome will be the proportion of eligible clients who receive a prescription through the CPA-supported workflow. These outcomes will provide evidence of whether CPA implementation supports PrEP initiation in community pharmacies located in high-HIV-prevalence neighborhoods in Georgia.

#### Planned Analyses

Phase 3 analyses will follow the hybrid type 1 effectiveness-implementation design by incorporating both implementation and effectiveness assessments. Qualitative data from pharmacist and clinician interviews will be analyzed using the reflexive thematic analysis, applying CFIR and SEIPS constructs to characterize facilitators and barriers of CPA adoption, workflow integration, and variations across pharmacies. Quantitative effectiveness analysis will be descriptive and will summarize client movement through the CPA-enabled PrEP pathway. We will examine site-level variation and assess changes in these indicators over the implementation period to understand how CPA integration influences PrEP initiation.

## Results

### Phase 1

Participant recruitment and data collection began in November 2023. As of January 5, 2026, a total of 264 participants have been recruited and have completed phase 1 pharmacy staff surveys, including 27 IDIs. Recruitment and data collection for phase 1 are anticipated to be completed by February 2026, with a target of 300 surveys completed and 68 IDI participants interviewed.

### Phase 2

We compiled and analyzed state-specific requirements for CPAs across CAP-UP states (Alabama, Florida, Georgia, Kentucky, Mississippi, North Carolina, South Carolina, and Tennessee) to develop a standardized yet adaptable CPA template. Data collection involved reviewing the Centers for Disease Control and Prevention CPA Toolkit, state pharmacy board regulations, and existing policies. CPA components were categorized into 3 main domains: scope of agreement, which defines the pharmacist’s clinical authority and prescriptive limitations; legal components, which outline regulatory requirements, including physician oversight and patient inclusion criteria; and administrative components, which detail documentation, data retention, and reporting obligations. Preliminary findings indicate that most states require a defined scope of agreement and prescriptive authority limitations, while supervision requirements and patient inclusion criteria vary, with some states mandating CPAs to name individual patients. Additionally, administrative requirements, such as data retention policies, range from 5 to 10 years. Phase 1 data will be used to refine the initial template, integrating stakeholder feedback to ensure it is tailored to the specific regulatory and operational contexts of each state. These findings will inform the development of a master CPA template with state-specific modifications, which is anticipated to be publicly available by 2026.

### Phase 3

Data collection began in winter 2025, and interim and final results are forthcoming.

## Discussion

### Implementation and Policy Implications

The CAP-UP study highlights the transformative potential of CPAs in addressing critical gaps in HIV prevention services, particularly in the Southeastern US, where inequities in HIV prevention access remain profound. By leveraging pharmacies as accessible, trusted points of care, CPAs can enhance access to PrEP for disproportionately affected populations, including Black communities and those in underserved areas [1,9,10,35]. This study’s use of the EPIS, CFIR, and SEIPS frameworks ensures a robust analysis of implementation barriers, facilitators, and operational needs, providing a clear pathway for scaling pharmacy-based PrEP services [63-65,73]. The hybrid type 1 design offers an opportunity to simultaneously evaluate implementation and effectiveness outcomes, generating evidence that can inform sustainable, real-world applications of CPAs [65,70]. However, regional policy constraints pose substantial challenges to this effort. Varying state regulations, limitations on pharmacist-prescribing authority, and the complexity of navigating collaborative agreements often hinder the widespread adoption of pharmacy-based PrEP services [61,70,71]. These barriers highlight the urgent need for policy reforms to empower pharmacists and streamline CPA implementation as well as the integration of HIV prevention services via pharmacist-led services [54,55,71]. Until legislation is fully expanded to allow pharmacists prescription authority for PrEP, developing and piloting adaptable CPA templates may create a scalable framework that can be applied across diverse pharmacy settings to enhance PrEP access [56,63,70]. Moreover, the CAP-UP study could inform advocacy for policy changes that empower pharmacists to operate at a higher level, reducing barriers to CPA implementation nationwide [56,71]. This includes addressing state-specific restrictions and fostering collaboration between regulatory boards and policymakers to streamline approval processes [56,71,72]. These advancements have the potential to not only reduce new HIV infections but also strengthen the role of pharmacies as essential health care hubs in underserved communities [7,34-42]

### Limitations

The CAP-UP study has several limitations that should be considered when interpreting its findings. Although efforts are made to recruit an adequate sample of pharmacy staff from each state, the results may not fully represent the experiences and practices of all pharmacies across the US Southeast. Voluntary participation may introduce selection bias, and variability in pharmacy workflows and clinician engagement could influence implementation outcomes. Although the CPA template is designed to be adaptable, significant modifications may be required in certain settings, reducing its potential for standardization. Regulatory barriers specific to the region and stigma surrounding HIV prevention could hinder engagement from both pharmacies and clients. Self-reported data may be affected by recall and social desirability biases, and cost estimates based on assumptions about pharmacists’ time may not reflect actual expenses in all contexts. Limited training, resources, and access to technology may further impede participation and implementation, while delays in recruitment and data collection could disrupt the study timeline.

### Conclusions

Despite these limitations, the CAP-UP study addresses critical gaps in access to HIV prevention services by leveraging pharmacist-clinician CPAs to expand pharmacy-based PrEP services in the US Southeast. This approach focuses on improving access for high-risk populations, particularly in communities where systemic barriers and stigma have historically limited health care access. Through the use of a hybrid type 1 design, the study systematically evaluates the barriers and facilitators to implementing CPAs, develops a flexible CPA template tailored to diverse pharmacy settings, and pilots the integration of CPAs in community pharmacies serving high-risk populations. Ultimately, the findings will inform future efforts to integrate pharmacists into HIV prevention strategies, contributing to the goal of reducing inequities and curbing the HIV epidemic in disproportionately affected communities.

## Abbreviations

CAP-UP: Collaborative Agreement–based PrEP Using Pharmacies
CFIR: Consolidated Framework for Implementation Research
CPA: collaborative practice agreement
EHE: Ending the HIV Epidemic
EPIS: Exploration, Preparation, Implementation, Sustainment
IDI: in-depth interview
PnR: PrEP-to-need ratio
PrEP: pre-exposure prophylaxis
SEIPS: Systems Engineering Initiative for Patient Safety
US: United States
